# The Role of Adipose Tissue Mitochondria: Regulation of Mitochondrial Function for the Treatment of Metabolic Diseases

**DOI:** 10.3390/ijms20194924

**Published:** 2019-10-04

**Authors:** Jae Ho Lee, Anna Park, Kyoung-Jin Oh, Sang Chul Lee, Won Kon Kim, Kwang-Hee Bae

**Affiliations:** 1 Metabolic Regulation Research Center, Korea Research Institute of Bioscience and Biotechnology (KRIBB), Daejeon 34141, Korea; 2 Department of Functional Genomics, KRIBB School of Bioscience, Korea University of Science and Technology (UST), Daejeon 34141, Korea

**Keywords:** mitochondria, mitochondrial dysfunction, white adipose tissue (WAT), brown adipose tissue (BAT), thermogenesis, browning, metabolic diseases

## Abstract

Mitochondria play a key role in maintaining energy homeostasis in metabolic tissues, including adipose tissues. The two main types of adipose tissues are the white adipose tissue (WAT) and the brown adipose tissue (BAT). WAT primarily stores excess energy, whereas BAT is predominantly responsible for energy expenditure by non-shivering thermogenesis through the mitochondria. WAT in response to appropriate stimuli such as cold exposure and β-adrenergic agonist undergoes browning wherein it acts as BAT, which is characterized by the presence of a higher number of mitochondria. Mitochondrial dysfunction in adipocytes has been reported to have strong correlation with metabolic diseases, including obesity and type 2 diabetes. Dysfunction of mitochondria results in detrimental effects on adipocyte differentiation, lipid metabolism, insulin sensitivity, oxidative capacity, and thermogenesis, which consequently lead to metabolic diseases. Recent studies have shown that mitochondrial function can be improved by using thiazolidinedione, mitochondria-targeted antioxidants, and dietary natural compounds; by performing exercise; and by controlling caloric restriction, thereby maintaining the metabolic homeostasis by inducing adaptive thermogenesis of BAT and browning of WAT. In this review, we focus on and summarize the molecular regulation involved in the improvement of mitochondrial function in adipose tissues so that strategies can be developed to treat metabolic diseases.

## 1. Introduction

In 1890, Altmann identified ubiquitously existing cytoplasmic structures with genetic and metabolic autonomy and called them “bioblasts.” He proposed that these structures are “elementary organisms” resembling bacteria [1]. The term “mitochondria” was introduced in 1898 by Benda; this term originated from the Greek words “mitos” and “condros,” which meant thread granules [2]. Later, in the 1930s, Keilin proposed the idea of a “respiratory chain” and Warburg won the Nobel Prize for his discovery of the respiratory enzyme [3,4]. Further, Krebs and Johnson elucidated the citric acid cycle, and they were awarded the Nobel Prize for their contribution in 1953 [5]. However, until then, mitochondria were not at the center of these important discoveries. At that point, Albert Claude introduced a technique for isolating mitochondria, and he subsequently revealed that the major enzymes related to the citric acid cycle, fatty acid oxidation, and oxidative phosphorylation (OXPHOS) are exclusively present in mitochondria [6,7]. These studies show that mitochondria are the essential intracellular organelles for energy metabolism because there are unique enzymes and systems that convert the chemical energy derived from carbohydrates, lipids, and proteins into adenosine triphosphate (ATP) in the form of available energy for cells [8,9]. In addition, eukaryotic cells have the ability to initiate adaptive responses to different environmental stimuli such as cell growth, differentiation, death, and stress during energy demands. Mitochondria play an important role in various processes including energy homeostasis, apoptosis, autophagy, and inflammatory pathways by regulating their number or their morphology or by remodeling their organization and distribution [10,11,12].

Adipose tissue is the major organ that controls the overall energy homeostasis in a living organism. Briefly, in excess energy conditions, the adipose tissue stores the superabundant nutrients in the form of triglycerides, whereas during scarcity of energy, it supplies the nutrients to other tissues through lipolysis [13]. In mammals, there are two different types of adipose tissues, namely, white adipose tissue (WAT) and brown adipose tissue (BAT). Interestingly, WAT and BAT have opposite functions. The WAT stores excess energy as triglycerides, while the BAT is specialized in the dissipation of energy through heat production for maintaining the body temperature and energy consumption. Recent studies have demonstrated that beige adipocytes sporadically reside with white adipocytes and emerge in response to certain environmental cues. Beige adipocytes have the antagonistic functions of WAT and agonistic functions of BAT at the same time. In the basal state, beige adipocytes act as white adipocytes; however, when given signals that require heat production through energy consumption such as cold stimulation, these cells demonstrate BAT-like function and morphology [14].

It has been established that mitochondria in many tissues, including skeletal muscle, liver, and heart play a critical role in the regulation of systemic energy homeostasis [15]. Consistent with that in other tissues, the mitochondria of adipose tissues function as a provider of cellular energy, but they also have adipocyte-specific functions. Several studies have suggested that mitochondria in the adipose tissue are intimately integrated with critical adipocyte biology such as adipogenesis, lipid metabolism, and thermogenesis [16,17]. Furthermore, recent studies have revealed that mitochondria in adipocytes might play substantial roles in the regulation of whole-body energy homeostasis, control of insulin sensitivity and glucose metabolism, or crosstalk between muscles and adipose tissues [18,19]. In this review, we focus on the adipocyte-specific roles of mitochondria and discuss the molecular regulation to improve mitochondrial function in adipose tissues in view of treatment of obesity and metabolic diseases, which are the major health challenges worldwide.

## 2. Mitochondrial Properties and Adipocytes

### 2.1. Mitochondria in White Adipocytes

White adipocytes are spherical cells of various sizes depending on the size of a single lipid droplet. A lipid droplet is composed of triglycerides and accounts for more than 90% of the cell volume. Mitochondria of white adipocytes are elongated, thin, and variable in amount (Table 1) [20]. Although the cell type-selective roles of mitochondria in regulating metabolic homeostasis have been discussed previously [17], the specific functions of mitochondria in white adipocytes have not been characterized in detail as well as that of the mitochondria in brown adipocytes.

The white adipocyte mitochondria represent the main source of ATP similar to that in other tissues, and they play critical roles in the biological processes of the adipocytes such as differentiation, lipogenesis, lipolysis, and fatty acid oxidation [16,23]. One of the features of WAT is that it has fewer mitochondria than BAT, with less expression of fatty acid oxidation-related enzymes such as acyl-CoA dehydrogenase, suggesting that the activity of fatty acid oxidation in WAT is lower than that in BAT (Table 1) [24,25,26,27]. To understand the tissue-specific functions of mitochondria, Forner and colleagues compared in vivo mouse mitochondrial proteomes between white and brown fat cells [22]. The result of their analysis demonstrated significant qualitative and quantitative differences in the mitochondrial proteins in the two different types of adipose tissues. The mitochondria of brown fat cells are more similar to those of skeletal muscles, whereas those in white fat cells not only selectively express proteins that support lipogenic function but also degrade xenobiotics, thereby revealing the protective function of this tissue. In particular, the Molybdenum cofactor sulfurase C-terminal domain-containing protein 1 (Mosc1), a component of the prodrug-converting complex, and Acyl-coenzyme A synthase (Acsm5) with CoA ligase activity were exclusively detected in WAT (Table 1).

### 2.2. Mitochondria in Brown and Beige Adipocytes

In contrast to the white fat cells, the brown adipocytes have multilocular lipid droplets. Brown adipocyte mitochondria are morphologically different from white adipocyte mitochondria. These brown adipocyte mitochondria are apparently more numerous and larger and contain packed cristae compared to the mitochondria in white adipocytes (Table 1) [20]. The brown fat cells containing numerous mitochondria appear to be brownish in microscopic images because of the iron-containing heme cofactor in the mitochondrial enzyme, namely, cytochrome oxidase [28].

BAT specifically converts chemical energy into heat in adaptive thermogenesis. When activated by sympathetic stimulation, brown and beige adipocytes dissipate chemical energy stored in the form of triglycerides by lipolysis and fatty acid oxidation. Subsequently, the energy of the substrate oxidation is converted into heat [25,29,30]. This process, called the non-shivering thermogenesis of brown and activated beige adipocytes, is particularly important for small animals and infants who need greater heat production owing to the large surface-to-volume ratio and hibernation of animals [31]. The uncoupling protein-1 (UCP1), a mitochondrial protein, plays a major role in the thermogenic function of brown and activated beige adipocytes. The UCP1 is located in the inner membrane of the mitochondria, and it causes a proton leak across the inner membrane of the mitochondria, thereby converting the electrochemical energy into heat [32]. The UCP1-dependent heat production in brown and activated beige adipocytes is induced by fatty acids, which are generated by lipolysis upon thermogenic activation such as cold exposure. Further oxidation of acetyl-CoA, the end of β-oxidation, through the tricarboxylic acid (TCA) cycle and the electron transport chain provides energy to dissipate heat by UCP1 [33]. In contrast to UCP1, other UCPs such as UCP2 and UCP3 appear to be involved in the reduction of free radical levels rather than in adaptive thermogenesis [34].

Forner et al. demonstrated the mitochondrial feature of brown and activated beige adipocytes by proteomic analysis of mitochondria from mouse BAT and WAT [22]. Their findings showed that the proteins involved in pathways related to fatty acid metabolism, OXPHOS, and the TCA cycle were highly expressed in BAT compared to that in WAT. In addition, acetyl-CoA synthase 2-like (Acss1), which converts acetate to acetyl-CoA, and pyruvate dehydrogenase kinase 4 (Pdk4), which inhibits the pyruvate dehydrogenase complex, thereby reducing the conversion of pyruvate to acetyl-CoA, were detected only in BAT.

## 3. Role of the Mitochondria in Adipocytes

### 3.1. Adipocyte Differentiation

**Figure 1:**
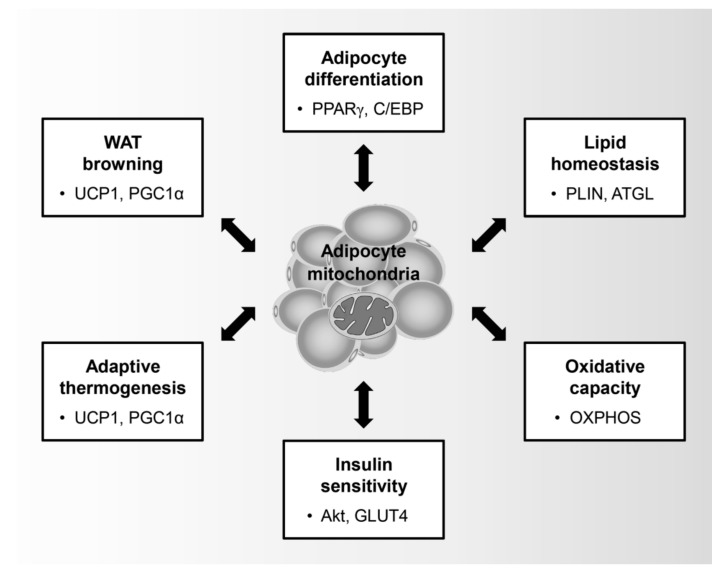
Physiological role of adipocyte mitochondria: Mitochondria in adipocytes regulate adipocyte differentiation, lipid homeostasis, oxidative capacity, insulin sensitivity, adaptive thermogenesis, and browning of white adipose tissues. Peroxisome proliferator-activated receptor-γ (PPARγ); CCAAT-enhancer-binding protein (C/EBP); Perilipin (PLIN); Adipose triglyceride lipase (ATGL); Oxidative phosphorylation (OXPHOS); Glucose transporter type 4 (GLUT4); Uncoupling protein-1 (UCP1); PPARγ coactivator 1-α (PGC1α).

Mitochondria are key organelles that control the physiological role of adipocytes such as adipocyte differentiation, lipid homeostasis, insulin sensitivity, oxidative capacity, adaptive thermogenesis, and browning of WATs (Figure 1). In particular, adipocyte differentiation is characterized by an induction in mitochondrial metabolism. During adipogenesis, preadipocytes undergo sequential transcriptional regulation by adipogenic regulators such as peroxisome proliferator-activated receptor-γ (PPARγ), CCAAT-enhancer-binding proteins (C/EBPs), and PPARγ coactivator 1-α (PGC1α), thereby leading to the promotion of mitochondrial biogenesis [35]. In addition, mitochondrial remodeling is involved in the quantitative and qualitative changes of the mitochondria during adipocyte differentiation [36]. Within 4 days of adipogenic induction of 3T3-L1, which are immortalized white preadipocytes, the expression of mitochondrial proteins and the number of mitochondria increases dramatically and is accompanied by qualitative changes in the mitochondrial composition, including pyruvate carboxylase and aconitase, which are involved in fatty acid metabolism [37]. Moreover, the increased oxygen consumption rate of the differentiated adipocytes compared to that of the preadipocytes is clear evidence that mitochondria are activated during adipocyte differentiation [38].

Previous studies have shown that the induction of mitochondria is essential for ATP production, which is necessary for increasing the metabolic activity during the adipogenic program. Despite promoted mitochondrial activity during adipocyte differentiation, the ATP content is decreased by high ATP consumption processes such as lipogenesis [37]. In the adipogenic program, the TCA cycle produces reducing equivalents such as nicotinamide adenine dinucleotide and flavin adenine dinucleotide and achieves oxidation of the acetyl-coenzyme A—an important metabolite produced from the catabolism of glucose and fatty acids [21]. Recently, it was demonstrated that mitochondrial dysfunction adversely affects adipocyte differentiation. Specific inhibitors of mitochondrial di- and tricarboxylate carriers prevent the differentiation of 3T3-L1 adipocytes, suggesting that mitochondria play an essential role in adipogenesis [39].

Mitochondrial reactive oxygen species (ROS) produced by the respiratory chain act as a second messenger and plays a crucial role in numerous cellular signaling pathways inside and outside the mitochondria. Mitochondrial ROS produced by OXPHOS complex III is essential for the initiation of adipocyte differentiation of human mesenchymal stem cells, whereas adipogenesis is inhibited by mitochondria-targeted antioxidants [23]. However, high doses of ROS negatively regulate the proliferation and differentiation of adipocytes [40]. In particular, increased ROS production by rotenone, a complex I inhibitor, or oligomycin, a F0-F1 ATP synthase inhibitor, has been reported to prevent the adipocyte differentiation of human mesenchymal stem cells [41,42]. A number of studies support the importance of mitochondria in adipocyte differentiation. Therefore, it is rational to target mitochondria for the treatment of obesity caused by the abnormal control of adipocyte differentiation.

### 3.2. Lipid Homeostasis and Oxidative Capacity

Adipose tissues play a key role in maintaining whole-body lipid homeostasis. Adipose tissues store lipids in the form of triglycerides in lipid droplets, thereby preventing ectopic lipid accumulation in other organs. Perilipin (PLIN), a lipid droplet coat protein, is required for the stable storage of lipid metabolites in adipocytes [43]. The triglycerides are broken into fatty acids and glycerol by adipose triglyceride lipase (ATGL)-mediated lipolysis, sequentially used as energy [44]. In obesity, excessive lipogenesis increases the total lipid pool, leading to insulin resistance and hyperglycemia [45]. It has been reported that the mitochondrial dysfunction with an OXPHOS complex III inhibitor causes triglyceride accumulation by inducing excessive lipogenesis in 3T3-L1 preadipocytes [46]. In particular, mitochondria in brown adipocytes are involved in lipid homeostasis by controlling fatty acid oxidation, which is one of the key pathways for lipid clearance [47]. Therefore, ectopic lipid accumulation is promoted by reducing fatty acid oxidation and energy expenditure due to mitochondrial dysfunction in the brown adipocytes of people with obesity and metabolic diseases [48].

A previous study showed that the abundance of mitochondrial populations is halved in adipocytes isolated from epididymal adipose tissues from obese and diabetic *ob*/*ob* mice [49]. Moreover, two obese and diabetic mouse models—the genetic model *db*/*db* mice and the dietary high-fat diet-fed mice—showed a decreased level of mitochondrial biogenesis regulators, including PGC1α and estrogen-related receptor-α, resulting in the loss of mitochondrial mass and structure [50]. Furthermore, mitochondrial dysfunction caused by tumor necrosis factor-α (TNF-α), which is an inflammatory cytokine that increases in obesity, leads to smaller and condensed mitochondria and inhibits intracellular ATP synthesis in 3T3-L1 adipocytes [51]. In addition, the expression of genes involved in OXPHOS complexes and fatty acid oxidation is decreased by TNF-α in primary adipocytes [52]. Under various pathological conditions, the oxidative capacity, biogenesis, density, and dynamics of mitochondria could be disrupted in the adipocytes, resulting in the development of obesity and metabolic diseases.

The main function of mitochondria in the adipocytes is to produce ATP to support a variety of metabolic pathways, including triglyceride synthesis, gluconeogenesis, and fatty acid re-esterification [21]. Dysregulation of mitochondria destroys the oxidative capacity and eventually fails to generate ATP. The mitochondrial membrane potential and the activity of the respiratory chain complexes are reported to be significantly decreased in the subcutaneous adipose tissues of obese subjects [53]. Moreover, the expression of the genes involved in mitochondrial oxidative pathways, including fatty acid oxidation, TCA cycle, ketogenesis, ketolysis, and branched chain amino acid degradation, is suppressed in obese individuals. One study showed that the protein levels of OXPHOS complexes III, IV, and V are also decreased in the adipose tissues of obese subjects [54]. Inverse correlations have been reported between body mass index and mitochondrial respiratory capacity in human adipose tissues. Furthermore, ADP-stimulated mitochondrial respiration, mtDNA copy number, and OXPHOS complex protein expression are dramatically decreased in the adipose tissues of obese subjects [55]. Thus, previous studies have shown the correlation between mitochondrial oxidative capacity in the adipocytes and systemic energy metabolism.

### 3.3. Glucose Utilization and Insulin Sensitivity

Insulin is a major anabolic hormone also involved in the regulation of energy and lipid metabolism through the phosphatidylinositol 3-kinase (PI3K)–Akt signaling pathway. The primary function of insulin is to allow glucose uptake into muscle cells and adipocytes to lower the blood glucose level. Binding of insulin to its receptor on adipocytes triggers the phosphorylation and activation of insulin receptor substrate (IRS), and it forms a docking site for PI3K at the cell membrane. When docked, PI3K converts phosphatidylinositol 4,5-bisphosphate (PIP_2_) to phosphatidylinositol 3,4,5-trisphosphate (PIP_3_). Subsequently PIP_3_ activates phosphoinositide-dependent protein kinase 1 (PDK1) and Akt. Akt stimulates translocation of glucose transporter type 4 (GLUT4) translocation to cell membranes, thus allowing glucose to enter the cell [56]. Adipocyte-specific *Glut4* knockout mice exhibit systemic glucose intolerance and insulin resistance [57]. 

Recently, mitochondria in adipocytes have been suggested to play a role in regulating insulin sensitivity. Treatments with respiratory inhibitors or uncoupling reagents that block mitochondrial function were shown to reduce insulin-stimulated glucose uptake in adipocytes [58]. In high-fat-diet-fed rats, mitochondrial biogenesis and the copy number of mitochondrial DNA (mtDNA) were decreased in epididymal adipose tissues, accompanied by hyperglycemia [59]. Further, high levels of glucose and/or free fatty acid attenuated insulin-stimulated glucose uptake by the dysfunctional mitochondria in the adipocytes. This glucose and/or free fatty acid-mediated reduction of glucose uptake was recovered by PGC1α overexpression, which is involved in the normalization of mitochondria [60]. The CR6-interacting factor-1 (Crif1) is a key translational factor responsible for the protein expression of mitochondrial OXPHOS complexes. *Crif1* ablation in adipocytes causes loss of OXPHOS complex subunits and respiratory complexes, sequentially resulting in whole-body insulin resistance [61]. These studies suggest that mitochondria in adipocytes are required for regulating glucose use through insulin signaling pathway.

### 3.4. Thermogenesis and WAT Browning

Recent studies have focused on the role of mitochondria in brown adipocytes that promotes energy consumption via adaptive thermogenesis [62]. Activation of mitochondria in brown adipocytes accelerates heat generation by increasing the inner mitochondrial membrane UCP1 [30]. Activated UCP1 uncouples the electron transport of the respiratory chain, thereby blocking ATP production and dissipating energy in the form of heat [63]. It has been demonstrated that the activity of UCP1 and thermogenesis in mouse BAT is associated with body-weight control and energy homeostasis [64]. Moreover, the adipocyte UCP1 expression is reduced in obese subjects, while metabolic complications are improved with UCP1 activation by various environmental and pharmacological stimuli [65]. Strategies for combating metabolic diseases through the activation of UCP1 have been proposed; for instance, eight subjects with diabetes reported a 43% increase in insulin sensitivity when UCP1 was activated by cold acclimation (14–15 °C) for 10 days [66]. Thus, adaptive thermogenesis-dependent fat burn has emerged as a plausible and safe strategy for relieving the metabolic syndrome.

The most important and well-studied factor that activates thermogenesis is norepinephrine (NE). NE affects cell proliferation and differentiation as well as thermogenesis of brown adipocytes. NE increases cyclic adenosine monophosphate levels via the β_3_-adrenergic receptor and activates protein kinase A, subsequently leading to lipolysis-mediated production of free fatty acids, one of the acute substrates of thermogenesis [67]. PGC1α has been reported to be essential for thermogenic activation by cold-induced and β_3_-adrenergic agonists in brown adipocytes [68]. PGC1α was first identified as a cold-induced interacting partner of PPARγ in BAT [69]. Mechanistically, PGC1α promotes mitochondrial biogenesis and oxidative metabolism by inducing UCP1 transcription [67]. Notably, thermogenic activation in brown adipocytes is accompanied by mitochondrial fission and depolarization [70]. These studies suggest that thermogenesis might be regulated by mitochondrial dynamics in brown adipocytes.

**Figure 2:**
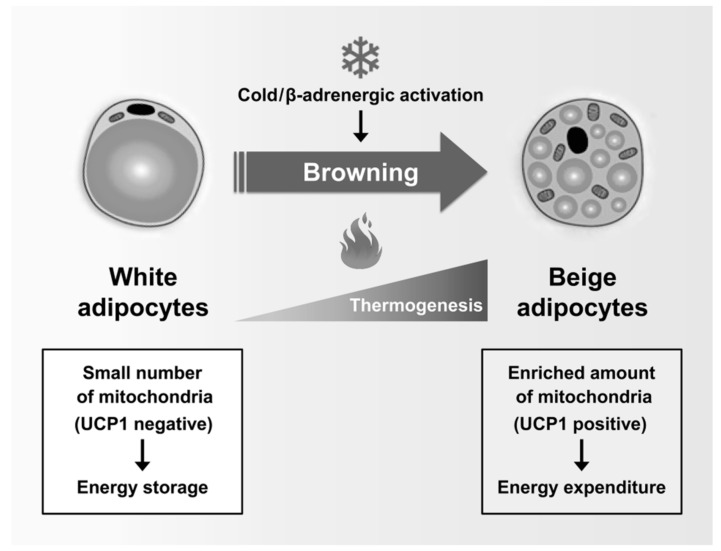
Regulation of adipose tissue browning: Beige adipocytes are generated by the browning of white adipose tissues in response to numerous stimuli, including cold exposure and activation of β-adrenergic receptors. Subsequently, the transcriptional machinery of the browning program activates the expression of characteristic thermogenic genes, leading to the formation of beige adipocytes. Similar to brown adipocytes, beige adipocytes are rich in mitochondria that express UCP1 and can achieve thermogenesis. Notably, beige adipocytes primarily contribute to energy expenditure rather than to energy storage.

A recent study showed that white adipocytes, which have low UCP1 expression and low mitochondrial contents, sometimes act as inducible thermogenic adipocytes that increase UCP1 expression and energy consumption under certain conditions such as cold exposure and β-adrenergic activation. Beige adipocytes (also called “brite”, “brown-like”, or “inducible brown”) are generated by the browning of WAT [71] (Figure 2). Beige adipocytes exhibit UCP1-dependent thermogenesis, and these mitochondria use lipids or carbohydrates as substrates, similar to brown adipocytes [72]. Chronic exercise decreases body weight and fat mass by inducing browning of subcutaneous WAT [73]. In addition, Irisin, a PGC1α-dependent myokine induced by exercise, has been shown to increase energy expenditure and to improve insulin sensitivity by activation of WAT browning [74]. In adults with low compositions of BAT, increased energy expenditure through WAT browning is an attractive strategy for preventing metabolic diseases. Taken together, mitochondria are essential organelles for maintaining the function of adipocytes in metabolic homeostasis.

## 4. Mitochondrial Dysfunction in Adipocytes

### 4.1. Increased Production of ROS

Mitochondrial dysfunction in adipocytes can affect whole-body energy dysregulation as well as insulin resistance. In addition, mitochondrial dysfunction has a significant association with obesity, which can eventually develop into metabolic diseases, including type 2 diabetes [75,76]. ROS might exhibit a beneficial or detrimental effect on the roles of adipocytes depending on the concentration and conditions. At moderate levels, ROS in the form of hydrogen peroxide plays an essential role in insulin signal transduction [75]. Kim et al. have provided evidence for the enhancement of insulin sensitivity by ROS in vivo [77]. Moreover, Tormos et al. suggested that the mechanistic target of rapamycin complex 1 (mTORC1)-promoted ROS is required for PPARγ-dependent adipogenesis during mesenchymal stem cell differentiation to adipocytes [23]. In addition, a recent study by Spiegelman et al. provided evidence for an important role of mitochondrial ROS in thermogenesis in brown and beige adipocytes [78]. They focused on the elevation of mitochondrial ROS during BAT thermogenesis and established that thermogenic ROS alters the redox status of cysteine thiols in BAT to drive increased respiration and that Cys-253 of UCP1 is a key target [78,79].

However, excessive mitochondrial ROS generation by chronic oxidative stress activates various stress pathways such as nuclear factor kappa light chain enhancer of activated B cells (NF-κB), c-Jun *N*-terminal kinase, and p38 mitogen-activated protein kinase to mediate immune and inflammatory response, leading to an increase in the expression of pro-inflammatory cytokines such as interleukin-6 (IL-6), TNF-α, and monocyte chemoattractant protein-1 (MCP-1) [80]. Subsequently, serine/threonine kinases are activated by these pro-inflammatory cytokines or ROS directly. The activated serine/threonine kinases increase the phosphorylation of the key components involved in the insulin signaling pathways such as insulin receptor (IR) and insulin receptor substrate (IRS). ROS-mediated phosphorylation of IR and IRS inhibits the recruitment and activation of the downstream Src Homology 2 (SH-2) containing signaling molecules and disrupt the ability of the IRS protein to interact with the IR [81,82,83]. Therefore, higher levels of ROS in mitochondria can contribute to the development of insulin resistance and the progression of various metabolic diseases, including type 2 diabetes. In addition, Wang et al. have reported that higher intracellular ROS levels result in the impairment of adipocyte function, which is accompanied by glucose intolerance and insulin resistance [58]. Given that mitochondria are important for adipocyte function, mitochondrial dysfunction caused by excessive ROS can lead to the impairment of adipogenesis and thermogenesis, which are the principal functions of adipocytes [78]. Despite many studies on the beneficial and detrimental effects of ROS in adipose tissues, further studies are required to elucidate the contribution of mitochondrial ROS in the roles of adipose tissue and metabolic homeostasis.

### 4.2. Alteration of the Mitochondrial Genome

Alteration of the mitochondrial genome can be more specifically divided into two types: reduction of the mitochondrial mass caused by the decrease of mtDNA related to mitochondrial biogenesis and mutation of mtDNA. Several investigations on obesity and type 2 diabetes have suggested that a decrease of mtDNA in the adipose tissues is associated with these diseases. For instance, mitochondrial mass and function are reduced in the WAT of *ob/ob* and *db/db* mice, which are the animal models of obesity [49,50,84]. Furthermore, Pietiläinen et al. have reported a decrease in the mtDNA of the WAT of obese human subjects [85].

Transcriptional coactivators PGC1α and PGC1β are known to be key components in the molecular network that coordinates the biogenesis of mitochondria in both WAT and BAT [69,86,87]. The fat-specific *Pgc1α*-deletion mice were more susceptible to glucose intolerance and insulin resistance than wild-type mice, and they displayed a decrease in uncoupling respiration. However, they showed normal mitochondrial content during treatment with PPARγ agonist rosiglitazone by inducing mitochondrial biogenesis [88]. One possible explanation for that is compensation by PGC1β because PGC1β is also upregulated by rosiglitazone [89]. To test this idea, Pardo et al. compared the effects of knockdown of PGC1α and PGC1β by using siRNA in rosiglitazone-treated 3T3-L1 adipocytes. They reported that only PGC1β knockdown prevented the induction of mitochondrial OXPHOS enzymes and mitochondrial respiration. This finding provides evidence that PGC1β, not PGC1α, regulates the mitochondrial gene expression in white adipocytes [87]. The mitochondrial transcription factor A (Tfam) is one of the major regulators of mitochondrial biogenesis. In adipocyte-specific *Tfam*-knockout mice, the activity of the proteins in complexes I, III, and IV was significantly decreased, which resulted in adipocyte death and inflammation in the WAT [18].

The mtDNA lacks the DNA repair mechanisms found in the nucleus, and mtDNA is subject damage from ROS released as a byproduct during OXPHOS. Therefore, the mutation rate in mtDNA is higher than that in nuclear DNA (nDNA) [90]. Several studies have shown that a certain polymorphism of mtDNA caused by mutations might be associated with obesity and lipid metabolism [91,92]. In addition, a previous study indicated that lipomas can be caused by a mutation in the mtDNA [93]. Therefore, it is plausible that the increase of ROS and mtDNA alteration can cause metabolic diseases by promoting mitochondrial dysfunction in adipocytes.

### 4.3. Dysregulated Mitochondrial Dynamics

Recent studies have indicated that mitochondrial structural remodeling through mitochondrial dynamics, including fusion and fission, is important for mitochondrial function [94,95]. In accordance with these, the imbalance of mitochondrial dynamics is associated with the pathophysiology of metabolic diseases such as obesity and type 2 diabetes [96]. Over the past several years, key regulators of mitochondrial dynamics have been identified under various conditions. The process of mitochondrial fusion is regulated by GTPase proteins, mitofusin 1 and 2 (MFN1/2) and optic atrophy 1 (OPA1), which are located in the outer and inner mitochondrial membranes, respectively [97,98]. Once intimate contact between mitochondria is established, the fusion of the outer mitochondrial membranes merges through the formation of a complex between MFN1 and MFN2 [99]. After tethering, the fusion of the inner mitochondrial membranes is mediated by OPA1 depending on the internal membrane potential [100]. The mitochondrial fusion process maintains the mitochondrial capacity and retains the genetic and biochemical homogeneity by allowing the dilution of increased ROS levels and mutated DNA and the repolarization of membranes [94]. Further, mitochondrial fusion is controlled by two highly conserved GTPases, fission 1 (FIS1) and dynamin-related protein 1 (DRP1), which are localized in the outer mitochondrial membrane and in the cytosol, respectively. The oligomerization of DRP1 forms a helical structure, and it hydrolyzes GTP and divides the mitochondria by constriction. In the fusion process, the mitochondrial content is mixed and the electrical conductivity is sustained throughout the mitochondria [101].

These mitochondrial dynamics are essential for preserving a healthy mitochondrial population. The remodeling of the mitochondrial structure is dynamic and sensitive to metabolic signals [102]. Mitochondrial fusion is positively involved in enhanced ATP production, while fission is associated with diminished OXPHOS, mtDNA depletion, and ROS generation. The balance of fusion and fission can be tilted in either direction depending on the changes in nutrient availability and metabolic demands, resulting in fragmented or hypertubular mitochondria [103]. Dysregulated mitochondrial dynamics adversely affect mitochondrial function, leading to excessive production of ROS, altered mitochondrial enzyme activity, impaired calcium homeostasis, reduced ATP production, and systemic energy abnormalities [104,105]. It has been reported that intracellular triglyceride accumulation is induced when the mitochondrial dynamics is destroyed by the knockdown of *Mfn2* or *Opa1* in 3T3-L1 adipocytes [106]. Moreover, adipocyte-specific *Mfn2*-deficient mice present severe structural abnormalities of the mitochondria and impaired adaptive thermogenesis [107,108]. In addition, mitochondrial fission by protein kinase A (PKA)-dependent phosphorylation of DRP1 at serine 600 is essential for the thermogenesis of the brown adipocytes, and impairment of this regulatory pathway disrupts brown adipocyte function [70]. It has been reported that reduced expression of MFN2 is associated with the reduced function of mitochondria in subcutaneous and visceral adipose tissues of obese subjects [109]. Furthermore, adipocyte-selective inducible deletion of *Mfn2* in mice leads to obesity and insulin resistance [109]. The role of mitochondrial dynamics in adipocyte metabolism has not been thoroughly elucidated, and further studies are required to understand the novel regulatory mechanisms of mitochondria in adipocytes.

### 4.4. Altered Mitophagy and Mitochondrial Turnover

Mitochondrial homeostasis is maintained by the balance between mitochondrial biogenesis and degradation. Mitochondrial degradation is carried out through autophagy—a breakdown process to remove and recycle unwanted or damaged cellular components in lysosomes [110]. Mitophagy is a quality control process of selectively clearing the excess mitochondria through autophagy when mitochondria have accumulated during adipogenesis or have been damaged by oxidative stress [111]. Mitophagy can be stimulated by Unc-51 like autophagy activating kinase 1 (ULK1) upon 5′-adenosine monophosphate-activated protein kinase (AMPK) activation or mTORC1 inhibition under cell maturation or nutritional deprivation [112]. The association between mitochondria and autophagosome, the initiation step of mitophagy, is mediated by the ubiquitin-dependent PTEN-induced kinase 1 (PINK1)-Parkin pathway [113]. Alternatively, mitochondria can be degraded by selective autophagy via microtubule-associated protein 1A/1B-light chain 3 (LC3) and p62 protein in an ubiquitin-independent manner [114]. In addition, mitochondria are removed by mitophagy to generate more lipid droplets by limiting fatty acid oxidation during adipocyte differentiation [114]. Recent studies on autophagy in adipocytes have provided insight into mitochondrial turnover and degradation pathways. Mice with adipocyte-specific inhibition of autophagy by ablation of autophagy-related gene 7 (*Atg7*) are resistant to diet-induced obesity and they exhibit improved insulin sensitivity, which is accompanied by the reduction of WAT mass but expansion of BAT [115,116]. The findings suggest that autophagy plays an important role in the clearance of mitochondria for adipocyte development.

Clinical studies have shown increased autophagy in the adipose tissues of human subjects with obesity and diabetes [117]. Increased autophagy under metabolic complications is the normal response for removing damaged mitochondria, which is induced by endoplasmic reticulum stress, oxidative stress, inflammation, and insulin resistance. Moreover, other clinical studies have shown higher accumulation of dysfunctional or metabolically impaired mitochondria in obese subjects than in lean groups [118,119]. These findings possibly suggest that mitophagy might be negatively controlled by excessive fat accumulation or obesity. In addition, mTORC1 activation and mitophagy inhibition increase the accumulation of damaged mitochondria in conditions of overnutrition [114]. Studies using autophagy-related gene knockout mice fed with a high-fat diet suggest that inhibition of lipogenesis and activation of mitophagy can occur as a compensatory mechanism when adipocyte autophagy is disrupted by malnutrition [116]. For instance, skeletal muscle-specific *Atg7* KO mice are resistant to diet-induced obesity, which are accompanied by induction of WAT browning [120]. Consistently, mitophagy contributes to whitening of brown and beige adipocytes, turning them into white adipocytes by mitochondrial clearance after withdrawal of β-adrenergic agonist or cold stimulus [121]. Moreover, it has been reported that mitophagy is required for mitochondrial homeostasis in BAT during cold challenge [122]. This ambivalence of mitophagy in adipocyte turnover and the presence of compensatory pathways with alternative autophagy may occur to effectively maintain the mitochondrial integrity and mass. In summary, mitophagy plays an essential role in eliminating impaired mitochondria in metabolic complications, but its effects in adipocytes is controversial because it is complicatedly regulated by various signals such as nutrition and stress conditions. Thus, further studies in this area will be needed to understand the physiological and the pathophysiological roles of mitophagy in adipocytes in metabolic diseases.

## 5. Targeting Mitochondria as a Therapeutic Strategy for Metabolic Diseases

### 5.1. Activation of BAT Thermogenesis and WAT Browning

Despite some understanding of the mechanisms of development and progression of obesity, it is still one of the major challenges in health worldwide. The current clinical approaches for the treatment of obesity include diet control, physical activity, drug therapy, and surgery [123]. Since recently applied anti-obesity therapies have shown several limitations and side effects, the regulation of mitochondrial activity in adipocytes has been proposed as a novel strategy. Mitochondrial activation in BAT has emerged as a safe prevention and management of obesity by increasing the energy expenditure. Cold exposure improves dyslipidemia and insulin resistance by accelerating plasma lipid clearance by triglyceride uptake into BAT in mice [124]. Evidences have shown the metabolically beneficial effect of BAT in genetic models with increased BAT [67] and transplantation of BAT [64]. In particular, BAT transplantation exhibits reduction of body weight and improvement in systemic insulin sensitivity [64]. Thus, increasing energy expenditure by BAT activation can be an appropriate strategy to overcome obesity.

Recently, WAT browning has been proposed as an alternative to BAT thermogenesis. Browning of WAT in many rodent models was found to result in resistance to diet-induced obesity and improvement in the systemic energy metabolism [125,126]. WAT browning has been reported to be induced by the chronic treatment of β_3_-adrenergic activators or PPARγ agonist thiazolidinedione (TZD) [127]. The browning process is regulated by various environmental factors such as hormones, chronic cold exposure, physical activity, and environmental enrichment [128]. Recent reports suggest that WAT browning by cold exposure or certain molecules such as Irisin and Neuregulin 4 may be an excellent strategy for controlling insulin-dependent diabetes [74,129]. Furthermore, fibroblast growth factor 21 (FGF21) potentiates cold-induced thermogenesis by promoting thermogenic gene expression and WAT browning [130]. Moreover, it has been revealed that beige adipocytes, which have similar characteristics to brown adipocytes, exist in humans [131]. In the last century, chemical uncoupler 2,4-dinitrophenol substances have been used to mimic the effects of activated UCP1, but when used at high doses, uncontrolled respiratory uncoupling resulted in side effects such as hyperthermia and death [67]. Therefore, further studies are needed to identify the applications of WAT browning without side effects to overcome metabolic diseases.

### 5.2. Thiazolidinediones

TZDs (thiazolidinediones), which are PPARγ agonists, belong to the class of antidiabetic drugs and are potent stimulators of adipocyte differentiation [132]. TZDs also affect many metabolic processes by regulating lipid mobilization, glycerol production, and gluconeogenesis in the liver and glucose use in the skeletal muscle and pancreatic beta cells, thereby contributing to the insulin-sensitizing effect [133]. In addition, treatment with TZDs promotes fatty acid oxidation by promoting the number of mitochondria in adipocytes, leading to systematic lipid homeostasis [134]. Rosiglitazone, a certain TZD, was found to dramatically improve insulin sensitivity, accompanied by mitochondrial biogenesis and remodeling, including shape and size in the white adipocytes of *ob*/*ob* mice. [49]. Another TZD, pioglitazone, is known to stimulate mitochondrial biogenesis and fatty acid oxidation by activating PGC1α in the subcutaneous adipose tissues of diabetic subjects [135]. Interestingly, it has been demonstrated that rosiglitazone enhances UCP1 expression and WAT browning in both rodents and humans [127]. TZD-mediated mitochondrial biogenesis and WAT browning are attractive pharmacological approaches, but further studies are required because the molecular mechanisms that control WAT browning in response to TZD are not thoroughly understood. Taken together, TZDs would be suitable applications to prevent or treat metabolic diseases by regulating mitochondrial function and energy expenditure.

### 5.3. Mitochondria-Targeted Antioxidants

Oxidative stress caused by mitochondrial dysfunction is known to be one of the etiologies of metabolic diseases. Thus, mitochondria-targeted antioxidants could be a potential therapy for metabolic and neurodegenerative diseases, which are associated with mitochondrial dysfunction. Many clinical studies have revealed that mitochondrial antioxidants such as vitamin E, *N*-acetylcysteine, glutathione, and coenzyme Q10 alleviate excessive ROS production and improve hyperglycemia in subjects with diabetes [136]. In 3T3-L1 adipocytes, treatment with *R*-α-lipoic acid, a mitochondria-targeted antioxidant, increases the oxygen consumption rate and fatty acid oxidation as well as promotes the expression of mitochondrial biogenesis-related genes such as PGC1α, mitochondrial transcription factor A, and nuclear respiratory factor-1 [137]. Moreover, other mitochondrial antioxidants such as ubiquinone (MitoQ) and vitamin E (MitoVitE) have been used to overcome mitochondrial dysfunction [138]. MitoQ is a potent mitochondria-specific antioxidant of which the lipophilic triphenylphosphonium (TPP) cation is bound to the ubiquinone antioxidant moiety of coenzyme Q10 [139]. The lipophilic TPP cation-mediated selective mitochondrial accumulation of MitoQ reduces mitochondrial ROS, which protects against mitochondrial defective diseases [140]. A recent study has shown that MitoQ mitigates metabolic diseases by regulating redox signaling pathways [141]. Similarly, SkQ1, which comprises a plastoquinone moiety, is also targeted by the mitochondria via the conjugation of the TPP cation, resulting in inhibition of mitochondrial ROS [142]. Another ROS scavenger mito-TEMPO, a mitochondria-targeted superoxide dismutase mimetic, improves insulin resistance and metabolic dysfunction [143]. These results support that excessive accumulation of mitochondrial ROS contributes to the development of metabolic diseases, implying that mitochondria-targeted antioxidants could alleviate metabolic complications.

However, previous studies has been demonstrated that the efficacy of antioxidants is low in the case of long-term supplementation. In patients with vascular diseases, antioxidant supplementation for several years did not prevented overall cardiovascular events and may increase the risk for heart failure [144]. In addition, long-term administration of the mitochondria-targeted antioxidants fails to attenuate oxidative damage in skeletal muscles of aged mice [145]. Thus, further studies are necessary to understand the different effects regarding the concentrations and duration of mitochondria-targeted antioxidant supplementation.

### 5.4. Exercise and Caloric Restriction

Mitochondrial abnormalities are involved in the development of metabolic and neurodegenerative diseases. Exercise increases systemic insulin sensitivity by improving mitochondrial biogenesis, respiration, content, and density in the skeletal muscles of individuals with diabetes [146]. Exercise mechanistically potentiates AMPK to induce PGC1α phosphorylation and activation, resulting in mitochondrial biogenesis [147]. Moreover, exercise stimulates Sirtuin 1 (SIRT1) deacetylase by increasing NAD^+^ content and leads to deacetylation-dependent PGC1α activation [148]. In addition to exercise, caloric restriction without malnutrition is a promising nongenetic and non-pharmacologic nutritional intervention that helps prolong life and prevent metabolic disease [149]. Caloric restriction has also been suggested to be an effective treatment for metabolic diseases by inducing mitochondrial biogenesis, oxygen consumption, ATP synthesis, and SIRT1-dependent PGC1α activation [150]. Recently, calorie restriction was reported to ameliorate hyperglycemia by increasing Akt2 activation and insulin-stimulated glucose uptake in the skeletal muscles of aged mice [151]. Taken together, exercise or calorie restriction improves whole-body metabolism through mitochondrial expansion.

### 5.5. Dietary Natural Compounds

In metabolic diseases, mitochondrial function and oxidative capacity are decreased in several metabolic tissues, including liver, skeletal muscle, and adipose tissues [152]. Recent studies have shown that a variety of dietary natural compounds such as polyunsaturated fatty acids and polyphenols could prevent and even treat metabolic diseases [153]. A variety of dietary polyunsaturated fatty acids improves insulin action on glucose use and prevents the development of whole-body insulin resistance [154]. For instance, α-linoleic acid, a plant-derived polyunsaturated fatty acid, has anti-obesity effects by promoting mitochondrial density and fatty acid oxidation in rodents and humans [155]. Dietary polyphenol resveratrol (3,5,4′-trihydroxystilbene) is mainly contained in grape skins and red wines. It is well known that resveratrol has antioxidant activities by reducing ROS production [156]. Furthermore, resveratrol increases systemic insulin sensitivity, mitochondrial biogenesis, and oxidative capacity by SIRT1-dependent PGC1α activation [157]. Consistently, other polyphenols, such as quercetin and hydroxytyrosol, have been shown to be very effective in inducing mitochondrial biogenesis through activating SIRT1-PGC1α pathway [158]. Recently, polyphenols have been reported to regulate energy metabolism by activating thermogenesis in BAT [159]. In addition, epigallocatechin-3-gallate (EGCG), which is abundant in green tea, has been reported to have antioxidant, anti-obesity, and anticancer activities [160]. Indeed, low doses of EGCG have been shown to improve insulin sensitivity via PGC1α activation in adipocytes [161]. EGCG is also involved in the induction of adaptive thermogenesis in humans [162]. Thus, various dietary natural compounds such as polyunsaturated fatty acids, polyphenols, and EGCG can improve insulin sensitivity by modulating mitochondrial function in the adipocytes.

## 6. Conclusions and Perspectives

**Figure 3:**
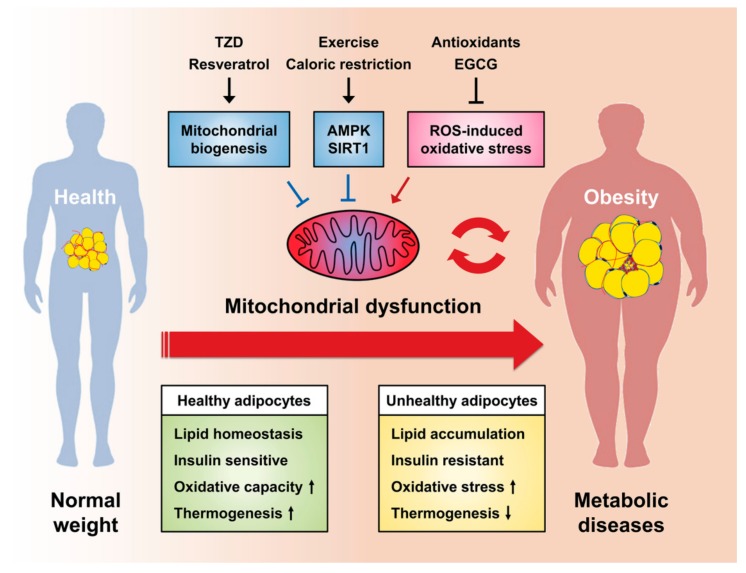
Therapeutic interventions for obesity and metabolic diseases by targeting mitochondrial regulation: Mitochondrial dysfunction due to impaired mitochondrial biogenesis and ROS -induced oxidative stress diminishes the role of the adipocytes. Therefore, mitochondrial dysfunction causes obesity and metabolic diseases, which in turn has a vicious cycle that leads to mitochondrial dysfunction. Recently, treatment of metabolic diseases has been suggested to involve induction of mitochondrial biogenesis in response to TZD and resveratrol treatment or by controlling mitochondrial function. In addition, mitochondria-targeted antioxidants and/or EGCG mitigate mitochondrial oxidative damage. Reactive oxygen species (ROS); Thiazolidinedione (TZD); Epigallocatechin-3-gallate (EGCG); 5’-Adenosine monophosphate-activated protein kinase (AMPK); Sirtuin 1 (SIRT1).

To date, the importance of mitochondria has been extensively investigated not only in energy supply but also in maintaining metabolic homeostasis. In particular, since adipocytes are metabolically active cells that require large amounts of ATP for regulating glucose and lipid metabolism, highly active mitochondria are crucial for adipocyte function. In adipocytes, mitochondria regulate adipocyte differentiation, lipid homeostasis, insulin sensitivity, oxidative capacity, thermogenesis, and WAT browning, while mitochondrial dysfunction causes a wide range of metabolic complications such as insulin resistance, obesity, and type 2 diabetes. Thus, understanding the molecular mechanisms of mitochondrial dysfunction of adipocytes implicated in the pathogenesis of metabolic diseases could provide potential opportunities for prevention and therapeutic intervention. Taken together, the improvement of the function of mitochondria by thermogenesis, PPARγ agonist TZD, mitochondria-targeted antioxidants, exercise, caloric restriction, and dietary natural compounds can be considered as a new potential therapeutic approach for the treatment of metabolic diseases (Figure 3). Effects of mitochondria-targeting drugs in adipocytes are quite promising; therefore, we hope that it will enable the development of effective metabolic disease therapies. Nonetheless, future studies are needed to elucidate the mechanisms underlying mitochondrial dysfunction and its pathogenic influence in the development of metabolic complications.

## Figures and Tables

**Table 1:** Mitochondrial characteristics of white and brown/beige adipocytes. Mitochondria (M); Nucleus (N); Lipid droplet (L); Pinocytotic vesicles (V); External basal membrane (MB); Molybdenum cofactor sulfurase C-terminal domain-containing protein 1 (Mosc1); Acyl-coenzyme A synthase (Acsm5); Acetyl-CoA synthase 2-like (Acss1); Pyruvate dehydrogenase kinase 4 (Pdk4).

Mitochondrial Property	White	Brown and Activated Beige	References
Morphology			[20]
(i) Size (ii) Shape (ii) Structure	Small Ellipsoid Elongated	Larger than white Spherical Packed cristae	
Content	Small number of mitochondria	Brown: Enriched Beige: Low to high upon stimulation	[21]
Development	Poorly developed	Highly developed	[21]
Major function	Differentiation Lipogenesis	Differentiation Fatty acid oxidation Thermogenesis	[22]
UCP1 expression	Low	High	[21]
Tissue-specific mitochondrial genes	Mosc1 Acsm5	Acss1 Pdk4	[22]

## References

[1] Altmann R. (1890). Die elementarorganismen und ihre beziehungen zu den zellen.

[2] Benda C. (1898). Ueber die Spermatogenese der Vertebraten und höherer Evertebraten, II Theil: Die Histiogenese der Spermien. Arch. Anat. Physiol..

[3] Warburg O. (1928). The Chemical Constitution of Respiration Ferment. Science.

[4] Dixon M., Keilin D. (1933). An improved method for the measurement of tissue respiration. Biochem. J..

[5] Krebs H.A. (1970). The history of the tricarboxylic acid cycle. Perspect. Biol. Med..

[6] Claude A., Fullam E.F. (1945). An Electron Microscope Study of Isolated Mitochondria: Method and Preliminary Results. J. Exp. Med..

[7] Ernster L., Schatz G. (1981). Mitochondria: A historical review. J. Cell Biol..

[8] Willis E.J. (1992). The powerhouse of the cell. Ultrastruct. Pathol..

[9] Pagliarini D.J., Rutter J. (2013). Hallmarks of a new era in mitochondrial biochemistry. Genes Dev..

[10] McBride H.M., Neuspiel M., Wasiak S. (2006). Mitochondria: more than just a powerhouse. Curr. Biol..

[11] Wang X. (2001). The expanding role of mitochondria in apoptosis. Genes Dev..

[12] Green D.R., Galluzzi L., Kroemer G. (2011). Mitochondria and the autophagy-inflammation-cell death axis in organismal aging. Science.

[13] Granneman J.G., Li P., Zhu Z., Lu Y. (2005). Metabolic and cellular plasticity in white adipose tissue I: Effects of beta3-adrenergic receptor activation. Am. J. Physiol. Endocrinol. Metab..

[14] Park A., Kim W.K., Bae K.H. (2014). Distinction of white, beige and brown adipocytes derived from mesenchymal stem cells. World J. Stem Cells.

[15] Johannsen D.L., Ravussin E. (2009). The role of mitochondria in health and disease. Curr. Opin. Pharmacol..

[16] Gregoire F.M., Smas C.M., Sul H.S. (1998). Understanding adipocyte differentiation. Physiol. Rev..

[17] Boudina S., Graham T.E. (2014). Mitochondrial function/dysfunction in white adipose tissue. Exp. Physiol..

[18] Vernochet C., Damilano F., Mourier A., Bezy O., Mori M.A., Smyth G., Rosenzweig A., Larsson N.G., Kahn C.R. (2014). Adipose tissue mitochondrial dysfunction triggers a lipodystrophic syndrome with insulin resistance, hepatosteatosis, and cardiovascular complications. FASEB J..

[19] Keuper M., Jastroch M., Yi C.X., Fischer-Posovszky P., Wabitsch M., Tschop M.H., Hofmann S.M. (2014). Spare mitochondrial respiratory capacity permits human adipocytes to maintain ATP homeostasis under hypoglycemic conditions. FASEB J..

[20] Cinti S. (2009). Transdifferentiation properties of adipocytes in the adipose organ. Am. J. Physiol. Endocrinol. Metab..

[21] De Pauw A., Tejerina S., Raes M., Keijer J., Arnould T. (2009). Mitochondrial (dys)function in adipocyte (de)differentiation and systemic metabolic alterations. Am. J. Pathol..

[22] Forner F., Kumar C., Luber C.A., Fromme T., Klingenspor M., Mann M. (2009). Proteome differences between brown and white fat mitochondria reveal specialized metabolic functions. Cell Metab..

[23] Tormos K.V., Anso E., Hamanaka R.B., Eisenbart J., Joseph J., Kalyanaraman B., Chandel N.S. (2011). Mitochondrial complex III ROS regulate adipocyte differentiation. Cell Metab..

[24] Orava J., Nuutila P., Lidell M.E., Oikonen V., Noponen T., Viljanen T., Scheinin M., Taittonen M., Niemi T., Enerback S. (2011). Different metabolic responses of human brown adipose tissue to activation by cold and insulin. Cell Metab..

[25] Cannon B., Nedergaard J. (2004). Brown adipose tissue: function and physiological significance. Physiol. Rev..

[26] Yehuda-Shnaidman E., Buehrer B., Pi J., Kumar N., Collins S. (2010). Acute stimulation of white adipocyte respiration by PKA-induced lipolysis. Diabetes.

[27] Rosell M., Kaforou M., Frontini A., Okolo A., Chan Y.W., Nikolopoulou E., Millership S., Fenech M.E., MacIntyre D., Turner J.O. (2014). Brown and white adipose tissues: intrinsic differences in gene expression and response to cold exposure in mice. Am. J. Physiol. Endocrinol. Metab..

[28] Enerback S. (2009). The origins of brown adipose tissue. N. Engl. J. Med..

[29] Bartelt A., Heeren J. (2014). Adipose tissue browning and metabolic health. Nat. Rev. Endocrinol..

[30] Nicholls D.G., Locke R.M. (1984). Thermogenic mechanisms in brown fat. Physiol. Rev..

[31] Kajimura S., Saito M. (2014). A new era in brown adipose tissue biology: molecular control of brown fat development and energy homeostasis. Annu. Rev. Physiol..

[32] Nicholls D.G. (1977). Stoicheiometries of proton translocation by mitochondria. Biochem. Soc. Trans..

[33] Lowell B.B., Spiegelman B.M. (2000). Towards a molecular understanding of adaptive thermogenesis. Nature.

[34] Bouillaud F., Alves-Guerra M.C., Ricquier D. (2016). UCPs, at the interface between bioenergetics and metabolism. Biochim. Biophys. Acta.

[35] Rosen E.D., Spiegelman B.M. (2000). Molecular regulation of adipogenesis. Annu. Rev. Cell Dev. Biol..

[36] Forni M.F., Peloggia J., Trudeau K., Shirihai O., Kowaltowski A.J. (2016). Murine Mesenchymal Stem Cell Commitment to Differentiation Is Regulated by Mitochondrial Dynamics. Stem Cells.

[37] Wilson-Fritch L., Burkart A., Bell G., Mendelson K., Leszyk J., Nicoloro S., Czech M., Corvera S. (2003). Mitochondrial biogenesis and remodeling during adipogenesis and in response to the insulin sensitizer rosiglitazone. Mol. Cell Biol..

[38] Si Y., Palani S., Jayaraman A., Lee K. (2007). Effects of forced uncoupling protein 1 expression in 3T3-L1 cells on mitochondrial function and lipid metabolism. J. Lipid Res..

[39] Kajimoto K., Terada H., Baba Y., Shinohara Y. (2005). Essential role of citrate export from mitochondria at early differentiation stage of 3T3-L1 cells for their effective differentiation into fat cells, as revealed by studies using specific inhibitors of mitochondrial di- and tricarboxylate carriers. Mol. Genet. Metab..

[40] Boneh A. (2006). Regulation of mitochondrial oxidative phosphorylation by second messenger-mediated signal transduction mechanisms. Cell Mol. Life Sci..

[41] Carriere A., Carmona M.C., Fernandez Y., Rigoulet M., Wenger R.H., Penicaud L., Casteilla L. (2004). Mitochondrial reactive oxygen species control the transcription factor CHOP-10/GADD153 and adipocyte differentiation: A mechanism for hypoxia-dependent effect. J. Biol. Chem..

[42] Zhang Y., Marsboom G., Toth P.T., Rehman J. (2013). Mitochondrial respiration regulates adipogenic differentiation of human mesenchymal stem cells. PLoS ONE.

[43] Brasaemle D.L. (2007). Thematic review series: adipocyte biology. The perilipin family of structural lipid droplet proteins: stabilization of lipid droplets and control of lipolysis. J. Lipid Res..

[44] Duncan R.E., Ahmadian M., Jaworski K., Sarkadi-Nagy E., Sul H.S. (2007). Regulation of lipolysis in adipocytes. Annu. Rev. Nutr..

[45] Sun K., Kusminski C.M., Scherer P.E. (2011). Adipose tissue remodeling and obesity. J. Clin. Investig..

[46] Vankoningsloo S., Piens M., Lecocq C., Gilson A., De Pauw A., Renard P., Demazy C., Houbion A., Raes M., Arnould T. (2005). Mitochondrial dysfunction induces triglyceride accumulation in 3T3-L1 cells: role of fatty acid beta-oxidation and glucose. J. Lipid Res..

[47] Vamecq J., Dessein A.F., Fontaine M., Briand G., Porchet N., Latruffe N., Andreolotti P., Cherkaoui-Malki M. (2012). Mitochondrial dysfunction and lipid homeostasis. Curr. Drug Metab..

[48] Bournat J.C., Brown C.W. (2010). Mitochondrial dysfunction in obesity. Curr. Opin. Endocrinol. Diabetes Obes..

[49] Wilson-Fritch L., Nicoloro S., Chouinard M., Lazar M.A., Chui P.C., Leszyk J., Straubhaar J., Czech M.P., Corvera S. (2004). Mitochondrial remodeling in adipose tissue associated with obesity and treatment with rosiglitazone. J. Clin. Investig..

[50] Rong J.X., Qiu Y., Hansen M.K., Zhu L., Zhang V., Xie M., Okamoto Y., Mattie M.D., Higashiyama H., Asano S. (2007). Adipose mitochondrial biogenesis is suppressed in db/db and high-fat diet-fed mice and improved by rosiglitazone. Diabetes.

[51] Chen X.H., Zhao Y.P., Xue M., Ji C.B., Gao C.L., Zhu J.G., Qin D.N., Kou C.Z., Qin X.H., Tong M.L. (2010). TNF-alpha induces mitochondrial dysfunction in 3T3-L1 adipocytes. Mol. Cell Endocrinol..

[52] Dahlman I., Forsgren M., Sjogren A., Nordstrom E.A., Kaaman M., Naslund E., Attersand A., Arner P. (2006). Downregulation of electron transport chain genes in visceral adipose tissue in type 2 diabetes independent of obesity and possibly involving tumor necrosis factor-alpha. Diabetes.

[53] Chattopadhyay M., Guhathakurta I., Behera P., Ranjan K.R., Khanna M., Mukhopadhyay S., Chakrabarti S. (2011). Mitochondrial bioenergetics is not impaired in nonobese subjects with type 2 diabetes mellitus. Metabolism.

[54] Heinonen S., Buzkova J., Muniandy M., Kaksonen R., Ollikainen M., Ismail K., Hakkarainen A., Lundbom J., Lundbom N., Vuolteenaho K. (2015). Impaired Mitochondrial Biogenesis in Adipose Tissue in Acquired Obesity. Diabetes.

[55] Fischer B., Schottl T., Schempp C., Fromme T., Hauner H., Klingenspor M., Skurk T. (2015). Inverse relationship between body mass index and mitochondrial oxidative phosphorylation capacity in human subcutaneous adipocytes. Am. J. Physiol. Endocrinol. Metab..

[56] Taniguchi C.M., Emanuelli B., Kahn C.R. (2006). Critical nodes in signalling pathways: insights into insulin action. Nat. Rev. Mol. Cell Biol..

[57] Abel E.D., Peroni O., Kim J.K., Kim Y.B., Boss O., Hadro E., Minnemann T., Shulman G.I., Kahn B.B. (2001). Adipose-selective targeting of the GLUT4 gene impairs insulin action in muscle and liver. Nature.

[58] Wang C.H., Wang C.C., Huang H.C., Wei Y.H. (2013). Mitochondrial dysfunction leads to impairment of insulin sensitivity and adiponectin secretion in adipocytes. FEBS J..

[59] Sutherland L.N., Capozzi L.C., Turchinsky N.J., Bell R.C., Wright D.C. (2008). Time course of high-fat diet-induced reductions in adipose tissue mitochondrial proteins: potential mechanisms and the relationship to glucose intolerance. Am. J. Physiol. Endocrinol. Metab..

[60] Gao C.L., Zhu C., Zhao Y.P., Chen X.H., Ji C.B., Zhang C.M., Zhu J.G., Xia Z.K., Tong M.L., Guo X.R. (2010). Mitochondrial dysfunction is induced by high levels of glucose and free fatty acids in 3T3-L1 adipocytes. Mol. Cell Endocrinol..

[61] Ryu M.J., Kim S.J., Kim Y.K., Choi M.J., Tadi S., Lee M.H., Lee S.E., Chung H.K., Jung S.B., Kim H.J. (2013). Crif1 deficiency reduces adipose OXPHOS capacity and triggers inflammation and insulin resistance in mice. PLoS Genet..

[62] Wang W., Seale P. (2016). Control of brown and beige fat development. Nat. Rev. Mol. Cell Biol..

[63] Zafrir B. (2013). Brown adipose tissue: Research milestones of a potential player in human energy balance and obesity. Horm. Metab. Res..

[64] Stanford K.I., Middelbeek R.J., Townsend K.L., An D., Nygaard E.B., Hitchcox K.M., Markan K.R., Nakano K., Hirshman M.F., Tseng Y.H. (2013). Brown adipose tissue regulates glucose homeostasis and insulin sensitivity. J. Clin. Investig..

[65] Cypess A.M., Kahn C.R. (2010). Brown fat as a therapy for obesity and diabetes. Curr. Opin. Endocrinol. Diabetes Obes..

[66] Hanssen M.J., Hoeks J., Brans B., van der Lans A.A., Schaart G., van den Driessche J.J., Jorgensen J.A., Boekschoten M.V., Hesselink M.K., Havekes B. (2015). Short-term cold acclimation improves insulin sensitivity in patients with type 2 diabetes mellitus. Nat. Med..

[67] Harms M., Seale P. (2013). Brown and beige fat: development, function and therapeutic potential. Nat. Med..

[68] Uldry M., Yang W., St-Pierre J., Lin J., Seale P., Spiegelman B.M. (2006). Complementary action of the PGC-1 coactivators in mitochondrial biogenesis and brown fat differentiation. Cell Metab..

[69] Puigserver P., Wu Z., Park C.W., Graves R., Wright M., Spiegelman B.M. (1998). A cold-inducible coactivator of nuclear receptors linked to adaptive thermogenesis. Cell.

[70] Wikstrom J.D., Mahdaviani K., Liesa M., Sereda S.B., Si Y., Las G., Twig G., Petrovic N., Zingaretti C., Graham A. (2014). Hormone-induced mitochondrial fission is utilized by brown adipocytes as an amplification pathway for energy expenditure. EMBO J..

[71] Giralt M., Villarroya F. (2013). White, brown, beige/brite: different adipose cells for different functions?. Endocrinology.

[72] Shabalina I.G., Petrovic N., de Jong J.M., Kalinovich A.V., Cannon B., Nedergaard J. (2013). UCP1 in brite/beige adipose tissue mitochondria is functionally thermogenic. Cell Rep..

[73] De Matteis R., Lucertini F., Guescini M., Polidori E., Zeppa S., Stocchi V., Cinti S., Cuppini R. (2013). Exercise as a new physiological stimulus for brown adipose tissue activity. Nutr. Metab. Cardiovasc. Dis..

[74] Bostrom P., Wu J., Jedrychowski M.P., Korde A., Ye L., Lo J.C., Rasbach K.A., Bostrom E.A., Choi J.H., Long J.Z. (2012). A PGC1-alpha-dependent myokine that drives brown-fat-like development of white fat and thermogenesis. Nature.

[75] Wang C.H., Wang C.C., Wei Y.H. (2010). Mitochondrial dysfunction in insulin insensitivity: implication of mitochondrial role in type 2 diabetes. Ann. N.Y. Acad. Sci..

[76] Kim J.A., Wei Y., Sowers J.R. (2008). Role of mitochondrial dysfunction in insulin resistance. Circ. Res..

[77] Loh K., Deng H., Fukushima A., Cai X., Boivin B., Galic S., Bruce C., Shields B.J., Skiba B., Ooms L.M. (2009). Reactive oxygen species enhance insulin sensitivity. Cell Metab..

[78] Chouchani E.T., Kazak L., Spiegelman B.M. (2017). Mitochondrial reactive oxygen species and adipose tissue thermogenesis: Bridging physiology and mechanisms. J. Biol. Chem..

[79] Chouchani E.T., Kazak L., Jedrychowski M.P., Lu G.Z., Erickson B.K., Szpyt J., Pierce K.A., Laznik-Bogoslavski D., Vetrivelan R., Clish C.B. (2016). Mitochondrial ROS regulate thermogenic energy expenditure and sulfenylation of UCP1. Nature.

[80] Bloch-Damti A., Bashan N. (2005). Proposed mechanisms for the induction of insulin resistance by oxidative stress. Antioxid. Redox. Signal..

[81] Evans J.L., Goldfine I.D., Maddux B.A., Grodsky G.M. (2003). Are oxidative stress-activated signaling pathways mediators of insulin resistance and beta-cell dysfunction?. Diabetes.

[82] Frank G.D., Eguchi S., Motley E.D. (2005). The role of reactive oxygen species in insulin signaling in the vasculature. Antioxid. Redox. Signal..

[83] Kim J.K. (2006). Fat uses a TOLL-road to connect inflammation and diabetes. Cell Metab..

[84] Choo H.J., Kim J.H., Kwon O.B., Lee C.S., Mun J.Y., Han S.S., Yoon Y.S., Yoon G., Choi K.M., Ko Y.G. (2006). Mitochondria are impaired in the adipocytes of type 2 diabetic mice. Diabetologia.

[85] Pietilainen K.H., Naukkarinen J., Rissanen A., Saharinen J., Ellonen P., Keranen H., Suomalainen A., Gotz A., Suortti T., Yki-Jarvinen H. (2008). Global transcript profiles of fat in monozygotic twins discordant for BMI: pathways behind acquired obesity. PLoS Med..

[86] Scarpulla R.C. (2011). Metabolic control of mitochondrial biogenesis through the PGC-1 family regulatory network. Biochim. Biophys. Acta.

[87] Pardo R., Enguix N., Lasheras J., Feliu J.E., Kralli A., Villena J.A. (2011). Rosiglitazone-induced mitochondrial biogenesis in white adipose tissue is independent of peroxisome proliferator-activated receptor gamma coactivator-1alpha. PLoS ONE.

[88] Rong J.X., Klein J.L., Qiu Y., Xie M., Johnson J.H., Waters K.M., Zhang V., Kashatus J.A., Remlinger K.S., Bing N. (2011). Rosiglitazone Induces Mitochondrial Biogenesis in Differentiated Murine 3T3-L1 and C3H/10T1/2 Adipocytes. PPAR Res..

[89] Kleiner S., Mepani R.J., Laznik D., Ye L., Jurczak M.J., Jornayvaz F.R., Estall J.L., Chatterjee Bhowmick D., Shulman G.I., Spiegelman B.M. (2012). Development of insulin resistance in mice lacking PGC-1alpha in adipose tissues. Proc. Natl. Acad. Sci. USA.

[90] Kujoth G.C., Bradshaw P.C., Haroon S., Prolla T.A. (2007). The role of mitochondrial DNA mutations in mammalian aging. PLoS Genet..

[91] Okura T., Koda M., Ando F., Niino N., Tanaka M., Shimokata H. (2003). Association of the mitochondrial DNA 15497G/A polymorphism with obesity in a middle-aged and elderly Japanese population. Hum. Genet..

[92] Liguori R., Mazzaccara C., Pasanisi F., Buono P., Oriani G., Finelli C., Contaldo F., Sacchetti L. (2006). The mtDNA 15497 G/A polymorphism in cytochrome b in severe obese subjects from Southern Italy. Nutr. Metab. Cardiovasc. Dis..

[93] Larsson N.G., Tulinius M.H., Holme E., Oldfors A. (1995). Pathogenetic aspects of the A8344G mutation of mitochondrial DNA associated with MERRF syndrome and multiple symmetric lipomas. Muscle Nerve Suppl..

[94] Westermann B. (2010). Mitochondrial fusion and fission in cell life and death. Nat. Rev. Mol. Cell Biol..

[95] Wai T., Langer T. (2016). Mitochondrial Dynamics and Metabolic Regulation. Trends Endocrinol. Metab..

[96] Wada J., Nakatsuka A. (2016). Mitochondrial Dynamics and Mitochondrial Dysfunction in Diabetes. Acta Med. Okayama.

[97] Hales K.G., Fuller M.T. (1997). Developmentally regulated mitochondrial fusion mediated by a conserved, novel, predicted GTPase. Cell.

[98] Cipolat S., Martins de Brito O., Dal Zilio B., Scorrano L. (2004). OPA1 requires mitofusin 1 to promote mitochondrial fusion. Proc. Natl. Acad. Sci. USA.

[99] Koshiba T., Detmer S.A., Kaiser J.T., Chen H., McCaffery J.M., Chan D.C. (2004). Structural basis of mitochondrial tethering by mitofusin complexes. Science.

[100] Song Z., Ghochani M., McCaffery J.M., Frey T.G., Chan D.C. (2009). Mitofusins and OPA1 mediate sequential steps in mitochondrial membrane fusion. Mol. Biol. Cell.

[101] Elgass K., Pakay J., Ryan M.T., Palmer C.S. (2013). Recent advances into the understanding of mitochondrial fission. Biochim. Biophys. Acta.

[102] Benard G., Bellance N., James D., Parrone P., Fernandez H., Letellier T., Rossignol R. (2007). Mitochondrial bioenergetics and structural network organization. J. Cell Sci..

[103] Liesa M., Shirihai O.S. (2013). Mitochondrial dynamics in the regulation of nutrient utilization and energy expenditure. Cell Metab..

[104] Blake R., Trounce I.A. (2014). Mitochondrial dysfunction and complications associated with diabetes. Biochim. Biophys. Acta.

[105] Bhatti J.S., Bhatti G.K., Reddy P.H. (2017). Mitochondrial dysfunction and oxidative stress in metabolic disorders—A step towards mitochondria based therapeutic strategies. Biochim. Biophys. Acta Mol. Basis Dis..

[106] Kita T., Nishida H., Shibata H., Niimi S., Higuti T., Arakaki N. (2009). Possible role of mitochondrial remodelling on cellular triacylglycerol accumulation. J. Biochem..

[107] Boutant M., Kulkarni S.S., Joffraud M., Ratajczak J., Valera-Alberni M., Combe R., Zorzano A., Canto C. (2017). Mfn2 is critical for brown adipose tissue thermogenic function. EMBO J..

[108] Mahdaviani K., Benador I.Y., Su S., Gharakhanian R.A., Stiles L., Trudeau K.M., Cardamone M., Enriquez-Zarralanga V., Ritou E., Aprahamian T. (2017). Mfn2 deletion in brown adipose tissue protects from insulin resistance and impairs thermogenesis. EMBO Rep..

[109] Mancini G., Pirruccio K., Yang X., Bluher M., Rodeheffer M., Horvath T.L. (2019). Mitofusin 2 in Mature Adipocytes Controls Adiposity and Body Weight. Cell Rep..

[110] Kim I., Rodriguez-Enriquez S., Lemasters J.J. (2007). Selective degradation of mitochondria by mitophagy. Arch. Biochem. Biophys..

[111] Ashrafi G., Schwarz T.L. (2013). The pathways of mitophagy for quality control and clearance of mitochondria. Cell Death Differ..

[112] Kim J., Kundu M., Viollet B., Guan K.L. (2011). AMPK and mTOR regulate autophagy through direct phosphorylation of Ulk1. Nat. Cell Biol..

[113] Narendra D.P., Jin S.M., Tanaka A., Suen D.F., Gautier C.A., Shen J., Cookson M.R., Youle R.J. (2010). PINK1 is selectively stabilized on impaired mitochondria to activate Parkin. PLoS Biol..

[114] Altshuler-Keylin S., Kajimura S. (2017). Mitochondrial homeostasis in adipose tissue remodeling. Sci. Signal.

[115] Singh R., Xiang Y., Wang Y., Baikati K., Cuervo A.M., Luu Y.K., Tang Y., Pessin J.E., Schwartz G.J., Czaja M.J. (2009). Autophagy regulates adipose mass and differentiation in mice. J. Clin. Investig..

[116] Zhang Y., Goldman S., Baerga R., Zhao Y., Komatsu M., Jin S. (2009). Adipose-specific deletion of autophagy-related gene 7 (atg7) in mice reveals a role in adipogenesis. Proc. Natl. Acad. Sci. USA.

[117] Kovsan J., Bluher M., Tarnovscki T., Kloting N., Kirshtein B., Madar L., Shai I., Golan R., Harman-Boehm I., Schon M.R. (2011). Altered autophagy in human adipose tissues in obesity. J. Clin. Endocrinol. Metab..

[118] Kraunsoe R., Boushel R., Hansen C.N., Schjerling P., Qvortrup K., Stockel M., Mikines K.J., Dela F. (2010). Mitochondrial respiration in subcutaneous and visceral adipose tissue from patients with morbid obesity. J. Physiol..

[119] Chattopadhyay M., Khemka V.K., Chatterjee G., Ganguly A., Mukhopadhyay S., Chakrabarti S. (2015). Enhanced ROS production and oxidative damage in subcutaneous white adipose tissue mitochondria in obese and type 2 diabetes subjects. Mol. Cell Biochem..

[120] Kim K.H., Jeong Y.T., Oh H., Kim S.H., Cho J.M., Kim Y.N., Kim S.S., Kim D.H., Hur K.Y., Kim H.K. (2013). Autophagy deficiency leads to protection from obesity and insulin resistance by inducing Fgf21 as a mitokine. Nat. Med..

[121] Altshuler-Keylin S., Shinoda K., Hasegawa Y., Ikeda K., Hong H., Kang Q., Yang Y., Perera R.M., Debnath J., Kajimura S. (2016). Beige Adipocyte Maintenance Is Regulated by Autophagy-Induced Mitochondrial Clearance. Cell Metab..

[122] Lu Y., Fujioka H., Joshi D., Li Q., Sangwung P., Hsieh P., Zhu J., Torio J., Sweet D., Wang L. (2018). Mitophagy is required for brown adipose tissue mitochondrial homeostasis during cold challenge. Sci. Rep..

[123] Powell T.M., Khera A. (2010). Therapeutic approaches to obesity. Curr. Treat. Options Cardiovasc. Med..

[124] Bartelt A., Bruns O.T., Reimer R., Hohenberg H., Ittrich H., Peldschus K., Kaul M.G., Tromsdorf U.I., Weller H., Waurisch C. (2011). Brown adipose tissue activity controls triglyceride clearance. Nat. Med..

[125] Vegiopoulos A., Muller-Decker K., Strzoda D., Schmitt I., Chichelnitskiy E., Ostertag A., Berriel Diaz M., Rozman J., Hrabe de Angelis M., Nusing R.M. (2010). Cyclooxygenase-2 controls energy homeostasis in mice by de novo recruitment of brown adipocytes. Science.

[126] Seale P., Conroe H.M., Estall J., Kajimura S., Frontini A., Ishibashi J., Cohen P., Cinti S., Spiegelman B.M. (2011). Prdm16 determines the thermogenic program of subcutaneous white adipose tissue in mice. J. Clin. Investig..

[127] Petrovic N., Walden T.B., Shabalina I.G., Timmons J.A., Cannon B., Nedergaard J. (2010). Chronic peroxisome proliferator-activated receptor gamma (PPARgamma) activation of epididymally derived white adipocyte cultures reveals a population of thermogenically competent, UCP1-containing adipocytes molecularly distinct from classic brown adipocytes. J. Biol. Chem..

[128] Sidossis L., Kajimura S. (2015). Brown and beige fat in humans: thermogenic adipocytes that control energy and glucose homeostasis. J. Clin. Investig..

[129] Christian M. (2015). Transcriptional fingerprinting of “browning” white fat identifies NRG4 as a novel adipokine. Adipocyte.

[130] Fisher F.M., Kleiner S., Douris N., Fox E.C., Mepani R.J., Verdeguer F., Wu J., Kharitonenkov A., Flier J.S., Maratos-Flier E. (2012). FGF21 regulates PGC-1alpha and browning of white adipose tissues in adaptive thermogenesis. Genes Dev..

[131] Wu J., Bostrom P., Sparks L.M., Ye L., Choi J.H., Giang A.H., Khandekar M., Virtanen K.A., Nuutila P., Schaart G. (2012). Beige adipocytes are a distinct type of thermogenic fat cell in mouse and human. Cell.

[132] Hauner H. (2002). The mode of action of thiazolidinediones. Diabetes Metab. Res. Rev..

[133] Tonelli J., Li W., Kishore P., Pajvani U.B., Kwon E., Weaver C., Scherer P.E., Hawkins M. (2004). Mechanisms of early insulin-sensitizing effects of thiazolidinediones in type 2 diabetes. Diabetes.

[134] Hock M.B., Kralli A. (2009). Transcriptional control of mitochondrial biogenesis and function. Annu. Rev. Physiol..

[135] Bogacka I., Xie H., Bray G.A., Smith S.R. (2005). Pioglitazone induces mitochondrial biogenesis in human subcutaneous adipose tissue in vivo. Diabetes.

[136] Fridlyand L.E., Philipson L.H. (2006). Reactive species, cellular repair and risk factors in the onset of type 2 diabetes mellitus: review and hypothesis. Curr. Diabetes. Rev..

[137] Shen W., Liu K., Tian C., Yang L., Li X., Ren J., Packer L., Cotman C.W., Liu J. (2008). R-alpha-lipoic acid and acetyl-L-carnitine complementarily promote mitochondrial biogenesis in murine 3T3-L1 adipocytes. Diabetologia.

[138] Kelso G.F., Porteous C.M., Coulter C.V., Hughes G., Porteous W.K., Ledgerwood E.C., Smith R.A., Murphy M.P. (2001). Selective targeting of a redox-active ubiquinone to mitochondria within cells: antioxidant and antiapoptotic properties. J. Biol. Chem..

[139] Smith R.A., Porteous C.M., Coulter C.V., Murphy M.P. (1999). Selective targeting of an antioxidant to mitochondria. Eur. J. Biochem..

[140] Smith R.A., Murphy M.P. (2011). Mitochondria-targeted antioxidants as therapies. Discov. Med..

[141] Feillet-Coudray C., Fouret G., Ebabe Elle R., Rieusset J., Bonafos B., Chabi B., Crouzier D., Zarkovic K., Zarkovic N., Ramos J. (2014). The mitochondrial-targeted antioxidant MitoQ ameliorates metabolic syndrome features in obesogenic diet-fed rats better than Apocynin or Allopurinol. Free Radic. Res..

[142] Skulachev V.P. (2007). A biochemical approach to the problem of aging: “megaproject” on membrane-penetrating ions. The first results and prospects. Biochemistry (Mosc.).

[143] Jeong E.M., Chung J., Liu H., Go Y., Gladstein S., Farzaneh-Far A., Lewandowski E.D., Dudley S.C. (2016). Role of Mitochondrial Oxidative Stress in Glucose Tolerance, Insulin Resistance, and Cardiac Diastolic Dysfunction. J. Am. Heart Assoc..

[144] Lonn E., Bosch J., Yusuf S., Sheridan P., Pogue J., Arnold J.M., Ross C., Arnold A., Sleight P., Probstfield J. (2005). Effects of long-term vitamin E supplementation on cardiovascular events and cancer: A randomized controlled trial. JAMA.

[145] Sakellariou G.K., Pearson T., Lightfoot A.P., Nye G.A., Wells N., Giakoumaki I.I., Griffiths R.D., McArdle A., Jackson M.J. (2016). Long-term administration of the mitochondria-targeted antioxidant mitoquinone mesylate fails to attenuate age-related oxidative damage or rescue the loss of muscle mass and function associated with aging of skeletal muscle. FASEB J..

[146] Toledo F.G., Menshikova E.V., Ritov V.B., Azuma K., Radikova Z., DeLany J., Kelley D.E. (2007). Effects of physical activity and weight loss on skeletal muscle mitochondria and relationship with glucose control in type 2 diabetes. Diabetes.

[147] Jager S., Handschin C., St-Pierre J., Spiegelman B.M. (2007). AMP-activated protein kinase (AMPK) action in skeletal muscle via direct phosphorylation of PGC-1alpha. Proc. Natl. Acad. Sci. USA.

[148] Rodgers J.T., Lerin C., Haas W., Gygi S.P., Spiegelman B.M., Puigserver P. (2005). Nutrient control of glucose homeostasis through a complex of PGC-1alpha and SIRT1. Nature.

[149] Omodei D., Fontana L. (2011). Calorie restriction and prevention of age-associated chronic disease. FEBS Lett..

[150] Civitarese A.E., Carling S., Heilbronn L.K., Hulver M.H., Ukropcova B., Deutsch W.A., Smith S.R., Ravussin E., Team C.P. (2007). Calorie restriction increases muscle mitochondrial biogenesis in healthy humans. PLoS Med..

[151] Wang H., Arias E.B., Cartee G.D. (2016). Calorie restriction leads to greater Akt2 activity and glucose uptake by insulin-stimulated skeletal muscle from old rats. Am. J. Physiol. Regul. Integr. Comp. Physiol..

[152] Heilbronn L.K., Gan S.K., Turner N., Campbell L.V., Chisholm D.J. (2007). Markers of mitochondrial biogenesis and metabolism are lower in overweight and obese insulin-resistant subjects. J. Clin. Endocrinol. Metab..

[153] Keijer J., van Schothorst E.M. (2008). Adipose tissue failure and mitochondria as a possible target for improvement by bioactive food components. Curr. Opin. Lipidol..

[154] Lalia A.Z., Lanza I.R. (2016). Insulin-Sensitizing Effects of Omega-3 Fatty Acids: Lost in Translation?. Nutrients.

[155] Flachs P., Horakova O., Brauner P., Rossmeisl M., Pecina P., Franssen-van Hal N., Ruzickova J., Sponarova J., Drahota Z., Vlcek C. (2005). Polyunsaturated fatty acids of marine origin upregulate mitochondrial biogenesis and induce beta-oxidation in white fat. Diabetologia.

[156] De la Lastra C.A., Villegas I. (2007). Resveratrol as an antioxidant and pro-oxidant agent: mechanisms and clinical implications. Biochem. Soc. Trans..

[157] Lagouge M., Argmann C., Gerhart-Hines Z., Meziane H., Lerin C., Daussin F., Messadeq N., Milne J., Lambert P., Elliott P. (2006). Resveratrol improves mitochondrial function and protects against metabolic disease by activating SIRT1 and PGC-1alpha. Cell.

[158] Wood dos Santos T., Pereira Q.C., Teixeira L., Gambero A., Villena J.A., Ribeiro M.L. (2018). Effects of Polyphenols on Thermogenesis and Mitochondrial Biogenesis. Int. J. Mol. Sci..

[159] Andrade J.M., Frade A.C., Guimaraes J.B., Freitas K.M., Lopes M.T., Guimaraes A.L., de Paula A.M., Coimbra C.C., Santos S.H. (2014). Resveratrol increases brown adipose tissue thermogenesis markers by increasing SIRT1 and energy expenditure and decreasing fat accumulation in adipose tissue of mice fed a standard diet. Eur. J. Nutr..

[160] Moon H.S., Lee H.G., Choi Y.J., Kim T.G., Cho C.S. (2007). Proposed mechanisms of (-)-epigallocatechin-3-gallate for anti-obesity. Chem. Biol. Interact..

[161] Lee M.S., Lee S., Doo M., Kim Y. (2016). Green Tea (-)-Epigallotocatechin-3-Gallate Induces PGC-1alpha Gene Expression in HepG2 Cells and 3T3-L1 Adipocytes. Prev. Nutr. Food Sci..

[162] Yoneshiro T., Matsushita M., Hibi M., Tone H., Takeshita M., Yasunaga K., Katsuragi Y., Kameya T., Sugie H., Saito M. (2017). Tea catechin and caffeine activate brown adipose tissue and increase cold-induced thermogenic capacity in humans. Am. J. Clin. Nutr..

